# Intrinsic and Chemotherapeutic Stressors Modulate ABCC-Like Transport in *Trypanosoma cruzi*

**DOI:** 10.3390/molecules26123510

**Published:** 2021-06-09

**Authors:** Kelli Monteiro da Costa, Eduardo J. Salustiano, Raphael do Carmo Valente, Leonardo Freire-de-Lima, Lucia Mendonça-Previato, José Osvaldo Previato

**Affiliations:** 1 Laboratory of Glycobiology, Carlos Chagas Filho Institute of Biophysics, Federal University of Rio de Janeiro (UFRJ), Rio de Janeiro 21941-902, RJ, Brazil; salustiano@bioqmed.ufrj.br (E.J.S.); leolima@biof.ufrj.br (L.F.L.); previato@biof.ufrj.br (J.O.P.); 2 Nucleus of Multidisciplinary Research in Biology (Numpex-Bio), UFRJ Duque de Caxias Campus—Professor Geraldo Cidade, Federal University of Rio de Janeiro (UFRJ), Duque de Caxias 25250-470, RJ, Brazil; raphael.valente@xerem.ufrj.br

**Keywords:** ABC transporter, drug resistance, benznidazole, ceramide, hemin, thiol, oxidative stress, environmental stress

## Abstract

*Trypanosoma cruzi* is the etiologic agent for Chagas disease, which affects 6–7 million people worldwide. The biological diversity of the parasite reflects on inefficiency of benznidazole, which is a first choice chemotherapy, on chronic patients. ABC transporters that extrude xenobiotics, metabolites, and mediators are overexpressed in resistant cells and contribute to chemotherapy failure. An ABCC-like transport was identified in the Y strain and extrudes thiol-conjugated compounds. As thiols represent a line of defense towards reactive species, we aimed to verify whether ABCC-like transport could participate in the regulation of responses to stressor stimuli. In order to achieve this, ABCC-like activity was measured by flow cytometry using fluorescent substrates. The present study reveals the participation of glutathione and ceramides on ABCC-like transport, which are both implicated in stress. Hemin modulated the ABCC-like efflux which suggests that this protein might be involved in cellular detoxification. Additionally, all strains evaluated exhibited ABCC-like activity, while no ABCB1-like activity was detected. Results suggest that ABCC-like efflux is not associated with natural resistance to benznidazole, since sensitive strains showed higher activity than the resistant ones. Although benznidazole is not a direct substrate, ABCC-like efflux increased after prolonged drug exposure and this indicates that the ABCC-like efflux mediated protection against cell stress depends on the glutathione biosynthesis pathway.

## 1. Introduction

*Trypanosoma cruzi* is a flagellated protozoan responsible for causing the anthropozoonosis Chagas disease [1]. According to the World Health Organization, Chagas disease is a neglected tropical disease with an estimated 6–7 million infected people in the world with almost 65 million at risk of infection. The Pan American Health Organization defined the endemic area as from southern United States to southern Argentina and Chile, which pertains to a total of 21 countries [2]. The main form of transmission is through the insect vector, which is predominant in rural areas with rudimentary infrastructure, promoting a favorable environment for its reproduction as well as proximity to the wild cycle [3]. Alternatively, *T. cruzi* may be transmitted by the consumption of food contaminated with vector feces or with the secretion of infected mammals [4]; donation of blood, organs or tissues, especially in countries that do not screen samples for *T. cruzi* [5]; and during pregnancy [6]. Furthermore, sexual transmission [7] and different species as potential vectors [8] are possible routes for the spreading of disease mainly in non-endemic areas.

First-line treatment of Chagas disease is performed with benznidazole, for which success depends on the stage of the disease, the patient’s age, and on biochemical characteristics of the strains [9]. Benznidazole is a prodrug, which contains a nitro radical attached to an imidazole ring. As such, its trypanocidal effect is second to the activation by a NADH-dependent trypanosomal type I nitroreductase and results in the formation of the dialdehyde glyoxal, which forms adducts with several biomolecules and DNA in special [10]. *T. cruzi* strains show great discrepancy in susceptibility to chemotherapy, with resistance detected on wild-type strains or after prolonged treatment [11,12]. Resistance to benznidazole in *T. cruzi* is multifactorial and results from mechanisms involving pro-drug activation, defenses against free radicals and drug efflux [13].

The life cycle of *T. cruzi* is complex and involves the epimastigote and amastigote replicative stages in the invertebrate and vertebrate hosts, respectively, and the trypomastigote infective stage [3]. In the hematophagous invertebrate host, parasites must thrive in a pro-oxidant microenvironment caused by the degradation of blood cells and subsequent heme release [14]. Regardless of being a necessary cofactor, heme is cytotoxic due to the generation of reactive oxygen species (ROS) [15]. Even though most ROS are a consequence of cellular respiration, xenobiotics arise as an important source of oxidative stress, either releasing ROS by biotransformation or via the direct consumption of antioxidant defenses [16]. In addition, stress-inducing agents are able to affect sphingolipid metabolism and results in the accumulation of ceramides [17], a sphingolipid comprised of a sphingosine-related base linked to a fatty acid through an amide bond. Ceramides are ubiquitous in nature as components of cell membranes and as regulators of cell cycle, differentiation, cell senescence, and apoptosis [18]. The nature of ceramide-mediated responses suggests that these sphingolipids coordinate pathways responsive to intracellular stresses [19], since their production is sensitive to redox metabolism [20].

ABCC is an ABC active transporter subfamily and is studied in several organisms owing to their ability to extrude endobiotics or xenobiotics alone, in conjugation to, or in cotransport with phosphate, glucuronide, or glutathione [21]. Human ABCC subfamily is able to transport sphingolipids as sphingosine-1-phosphate [22], sphingomyelin, glucosylceramides [23], and exogenous ceramides [24]. Moreover, ABCC activity contributes to chemotherapy resistance phenotype in major protozoa including *Leishmania*, *Trypanosoma,* and *Plasmodium* species [25]. In *T. cruzi*, 27 ABC genes were identified in the genome, the first being named PGP1 and PGP2 by Dallagiovanna et al. [26,27]. Subsequently, it was observed that the PGP1 and PGP2 genes show great similarity with the ABCC6 and ABCC2 genes of *L. major* and *T. brucei*, respectively [28]. Additionally, *T. cruzi* Y strain showed efflux of thiol-conjugated compounds [29] in a similar mechanism to that performed by ABCC transporters present on humans [30] as well as on other trypanosomatids [25]. Considering the importance of oxidative stress to *T. cruzi* development and the crucial participation of ABC transporters on cellular detoxification, we investigated the participation of metabolites involved in cell stress pathways elicited by the microenvironment or by chemotherapy in the ABCC-like efflux as well as the importance of such activity for *T. cruzi* strains with diverse phenotypes of resistance to benznidazole.

## 2. Results

### 2.1. Glutathione Promotes CF Accumulation

**Figure 1:**
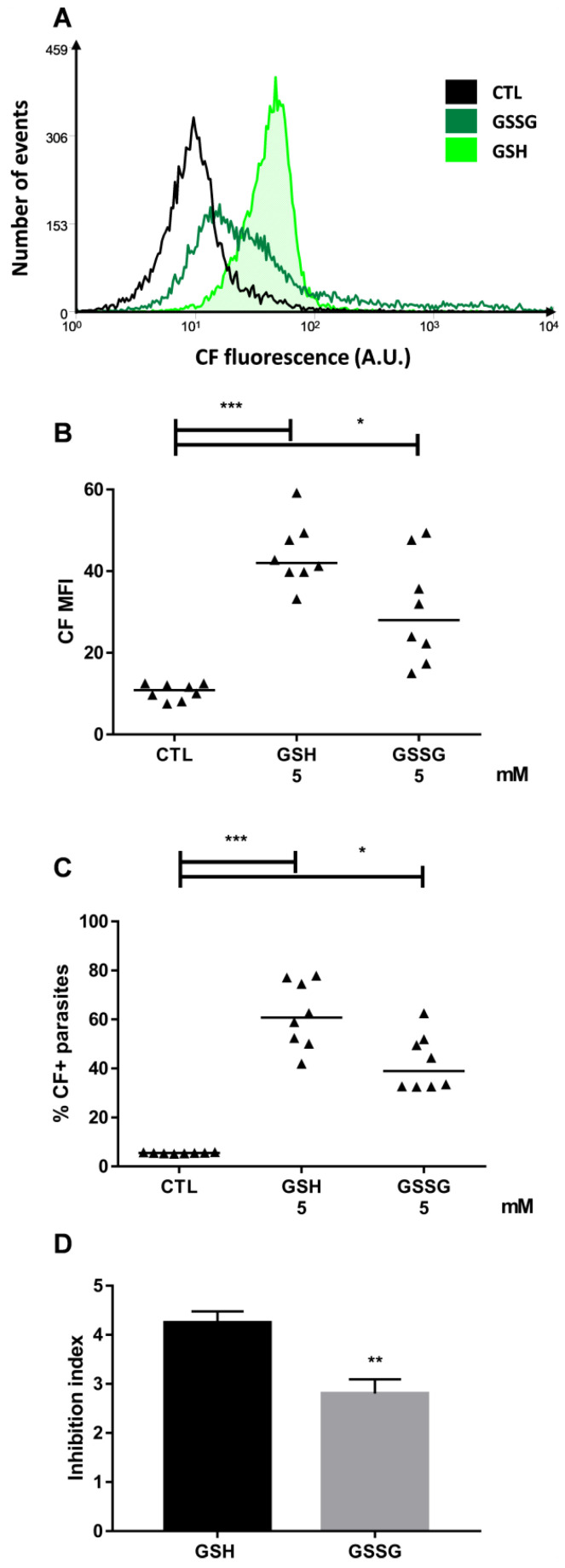
CF accumulation in the presence of glutathione. ABCC-like activity was evaluated by the CF efflux assay in the presence of 5 mM of GSH or GSSG. (**A**) Representative histograms, (**B**) CF MFI, (**C**) percentage of CF+ parasites, and (**D**) inhibition index compared to control (CTL) from the Y strain. Lines represent the median, bars represent mean + SEM, and the values of significance were represented by (*) for *p* < 0.05, (**) *p* < 0.01, and (***) *p* < 0.001, *n* = 8 independent experiments.

Carboxyfluorescein (CF) is a transportable fluorescent substrate employed in the ABCC efflux assay in the Y strain, as described in the Materials and Methods section. In this work, both forms of glutathione (reduced, GSH; oxidized, GSSG) increased the median fluorescence intensity (MFI) for CF and the percentage of CF-positive parasites compared to the control parasites (referred to as CTL) and this suggests that both molecules inhibited the CF efflux (Figure 1B,C). Representative histograms for these conditions are depicted in Figure 1A. Analyzing the transport inhibition index, which is calculated as the ratio of the CF MFI in the presence of GSH or GSSG to the one in its absence, it was observed that the reduced peptide inhibited the CF efflux more effectively than its oxidized version (Figure 1D).

### 2.2. Hemin Preincubation Promotes Thiol Depletion and CF Accumulation

**Figure 2:**
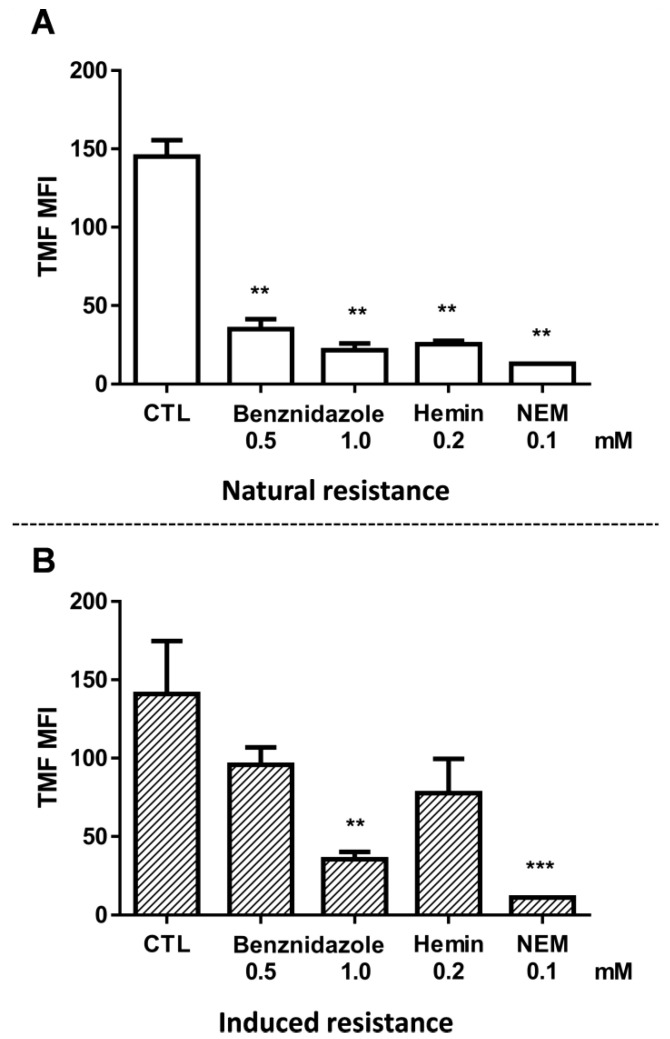
Intracellular levels of free thiols after benznidazole and hemin preincubation. A fluorescent probe was employed to indirectly measure the free thiol levels after 3 h preincubation with 0.5 mM or 1.0 mM benznidazole or 0.2 mM hemin. Bars represent the mean + SEM of thiol-conjugated methylfluorescein (TMF) MFI in the (**A**) Y strain and (**B**) Y-R^Bz^ parasites compared to respective controls (CTL). The values of significance were represented by (**) for *p* < 0.01 and (***) *p* < 0.001, *n* = 3 (Y) and *n* = 5 (Y-R^Bz^) independent experiments.

Hematophagous feeding of the invertebrate host results in ROS in the microenvironment where *T. cruzi* are found. In order to assess whether hemin is able to reduce the parasite’s antioxidant defenses, the levels of free thiols were measured using the fluorescent probe 5-chloromethylfluorescein diacetate (CMFDA) as described in the Materials and Methods section. Since CMFDA could also be a substrate of transporters of the ABCC subfamily [29], intracellular thiols were measured in the absence of glucose and cells were then maintained at 0 °C until the moment of acquisition. As such, this would drastically reduce the interference of ABCC-like transport. A 3 h preincubation with 0.2 mM hemin reduced thiol levels by 82.50% in the Y strain (Figure 2A); this is similar to the positive control *N*-ethylmaleimide (NEM) which is an alkylating agent. Free thiol levels were measured by thiol-conjugated methylfluorescein (TMF) MFI. In Figure 2B, the same methodology was employed to parasites resistant to benznidazole and denominated as Y-R^Bz^. The graph has been added here to facilitate comparison with the parental strain (Y strain). These results will be described in Section 2.6 CF Efflux Increases after Acquired Resistance to Benznidazole.

**Figure 3:**
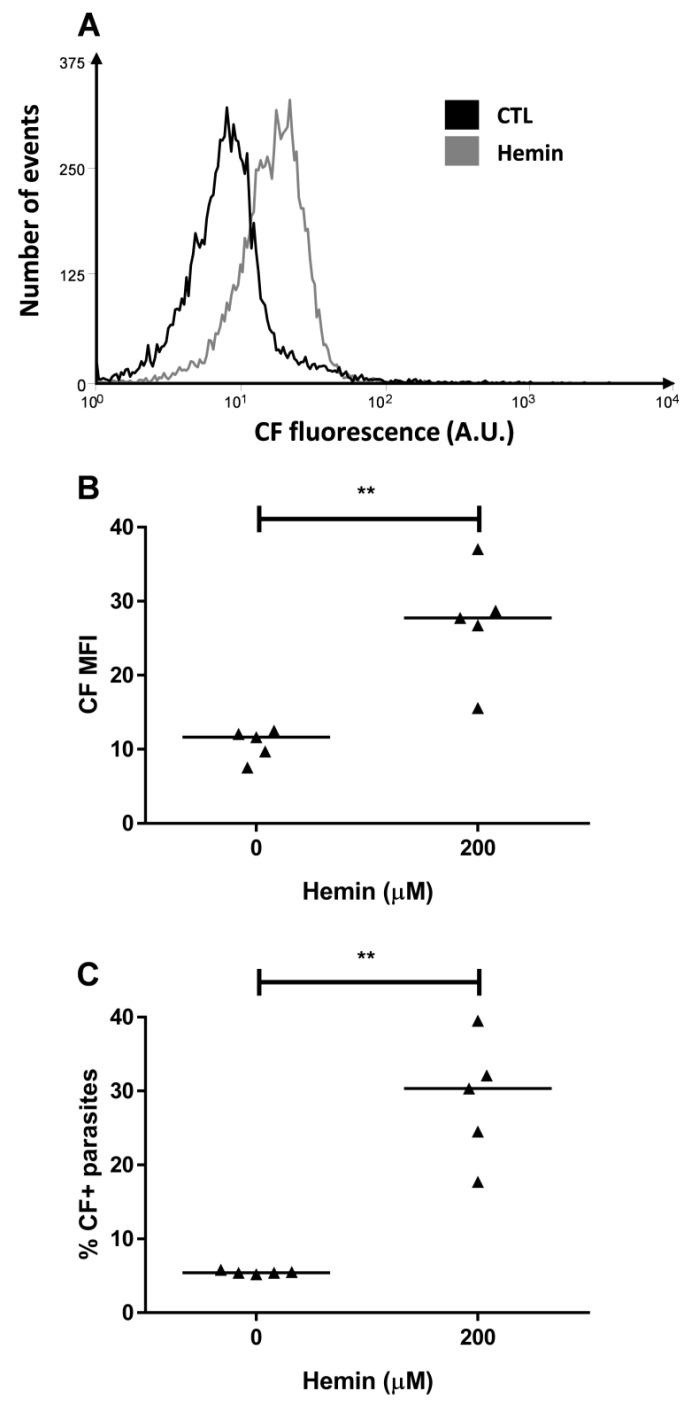
CF accumulation after hemin preincubation. ABCC-like activity was evaluated by CF efflux assay after a preincubation with 200 µM hemin for 1 h. (**A**) Representative histograms, (**B**) CF MFI and (**C**) percentages of CF+ parasites from Y strain. Lines represent the median and the values of significance represented by (**) *p* < 0.01, *n* = 5 independent experiments.

As thiol levels were reduced by hemin, we evaluated their effect on the ABCC activity. Hemin preincubation promoted increases in both CF MFI and in the percentage of CF-positive parasites (Figure 3), which suggests the participation of ABCC transporters in the detoxification pathways of the parasite.

### 2.3. ABCC-Like Activity Mediates Ceramide Efflux

**Figure 4:**
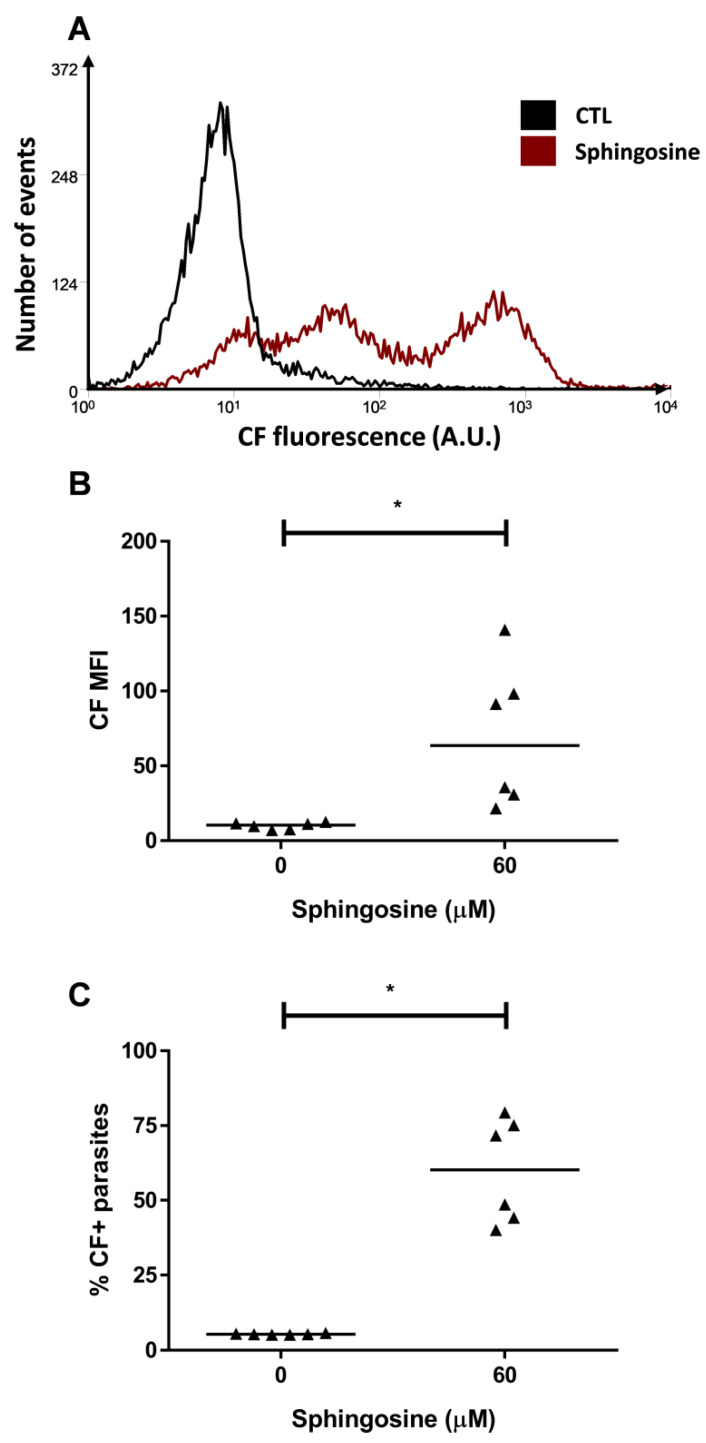
CF accumulation after sphingosine preincubation. ABCC-like activity was evaluated by the CF efflux assay after a preincubation with 60 µM sphingosine for 1 h. (**A**) Representative histograms, (**B**) CF MFI, and (**C**) percentages of CF+ parasites from Y strain. Lines represent the median and values of significance were represented by (*) for *p* < 0.05, *n* = 6 independent experiments.

Several approaches suggest sphingolipids might act as mediators of cellular responses to stress [19]. For this purpose, an 1 h preincubation was performed with 60 µM sphingosine, which is metabolized by ceramide synthase promoting the accumulation of ceramides [31]. In the Y strain, the preincubation with sphingosine increased CF MFI from 10.49 to 63.55 (Figure 4A,B). In addition, about 55% of the parasites accumulated CF in the cytosol (Figure 4C) in this condition.

**Figure 5:**
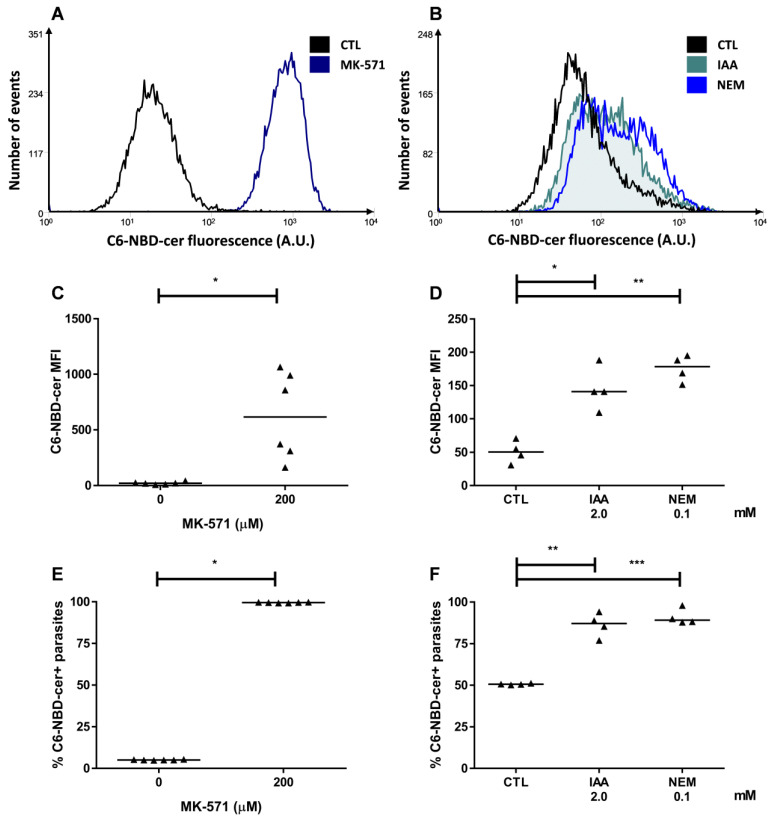
Ceramide efflux in the presence of ABCC inhibition and after ATP or thiol depletion. ABCC-like activity was evaluated by the C6-NBD-cer efflux assay in the presence of 200 µM MK-571 or a preincubation with 2 mM iodoacetic acid (IAA) or 100 µM N-ethylmaleimide (NEM) for 1 h. (**A**,**B**) Representative histograms, (**C**,**D**) 6-NBD-cer MFI, and (**E**,**F**) percentages of C6-NBD-cer+ parasites compared to the control (CTL) from Y strain. Lines represent the median and the values of significance were represented by (*) for *p* < 0.05, (**) *p* < 0.01, and (***) *p* < 0.001, *n* = 6 (MK-571) and *n* = 4 (IAA and NEM) independent experiments.

In order to rule out a possible interference of a direct transport of sphingosine, an ABCC-mediated efflux assay was carried out by using, as a substrate, a fluorescent analogue of ceramide, namely C6-NBD-cer (abbreviation to *N*-[(*E*,2*S*,3*R*)-1,3-dihydroxyoctadec-4-en-2-yl]-6-[(4-nitro-2,1,3-benzoxadiazol-7-yl)amino]hexanamide). Representative histograms for these conditions are depicted in Figure 5A,B. MK-571, a specific ABCC subfamily inhibitor, promoted an increase in C6-NBD-cer MFI with almost 100% of parasites inhibited (Figure 5C,E). Furthermore, iodoacetic acid (IAA) and NEM, ATP and thiol depletion agents, respectively, were employed prior to the efflux assay. It is noteworthy that both compounds induced C6-NBD-cer accumulation as observed by the increase in MFI and in the percentage of positive parasites (Figure 5D,F). The results showed that ABCC performs the efflux of short-chain ceramides and suggests that it would possibly transport sphingolipids produced during stress conditions.

### 2.4. Resistance to Benznidazole

In the present study, four *T. cruzi* strains were considered naturally susceptible or resistant to benznidazole. In order to confirm the susceptibility to the drug, epimastigote forms from Berenice, CL Brener, Y and Colombiana strains were treated with a range of concentrations of benznidazole for 48 h. Supplementary Figure S1A shows the half-maximum inhibitory concentration (IC_50_) to the four strains that was calculated by means of inhibition of mitochondrial reducing activity. Berenice and CL Brener strains are susceptible to treatment, with IC_50_ of 7.06 ± 1.16 µM and 10.42 ± 1.83 μM, respectively. The Y and Colombiana strains are significantly more resistant and presented IC_50_ of 31.02 ± 2.89 μM and 33.90 ± 1.41 μM, respectively.

Additionally, epimastigote forms from Y strain were selected in vitro after exposure to benznidazole as described in the Material and Methods section. This protocol produced great impact on adaptation to chemotherapy, since its IC_50_ reached 395.10 ± 35.84 μM (Supplementary Figure S1B). As described previously, the Y strain selected in vitro to benznidazole is referred to as Y-R^Bz^.

### 2.5. Natural Resistance to Benznidazole Does Not Relate to ABCC-Like Mediated Efflux

**Figure 6:**
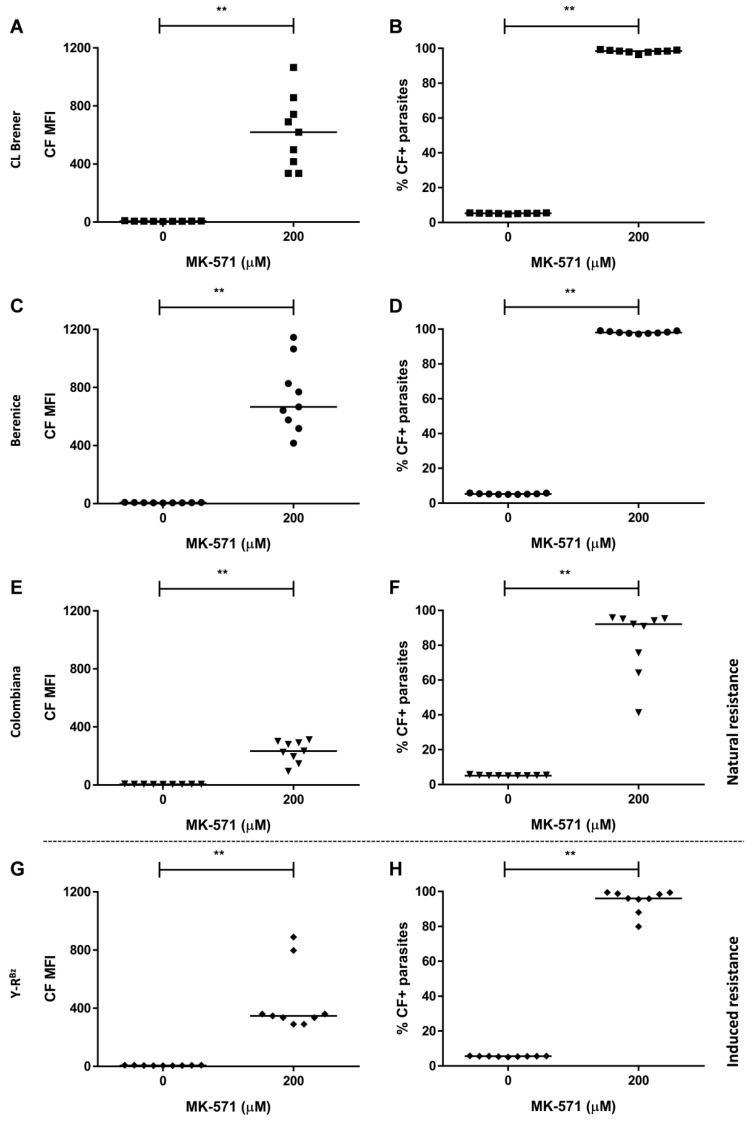
CF accumulation in the presence of the inhibitor MK-571 in *T. cruzi* sensitive and resistant to benznidazole. ABCC-like activity was evaluated by the carboxyfluorescein (CF) efflux assay in the presence of 200 μM MK-571. Graphs exhibit CF MFI (left panel) and percentages of CF+ parasites (right panel) from (**A**,**B**) CL Brener, (**C**,**D**) Berenice, (**E**,**F**) Colombiana strains, and (**G**,**H**) Y-R^Bz^ parasites. Lines represent the median and values of significance were represented by (**) for *p* < 0.01, *n* = 9 independent experiments.

Similar to hemin, benznidazole preincubation for 3 h reduced thiol levels by more than 70% in the Y strain (Figure 2A). In addition, the ABCC-like mediated efflux assay was performed in epimastigote forms of naturally resistant (Colombiana) or sensitive strains (Berenice and CL Brener) to the drug. CF MFI and percentages of CF-positive parasites were higher in the presence of the ABCC pharmacological inhibitors MK-571 (Figure 6A–F) and indomethacin (Supplementary Figure S2), which demonstrates that ABCC-mediated efflux is present in all strains.

**Figure 7:**
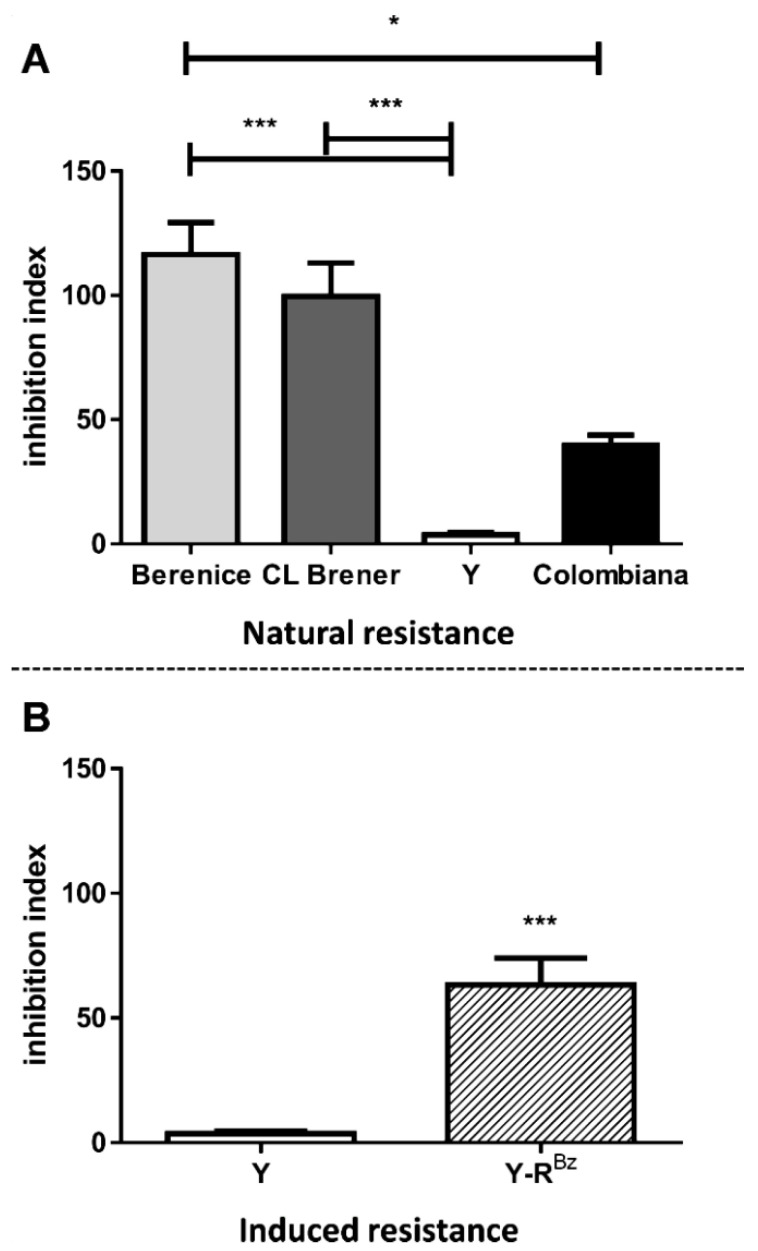
Inhibition indexes of CF efflux for MK-571 in *T. cruzi* strains. Inhibition indexes represent the ratio of the CF MFI in presence of 200 µM MK-571 to in the absence. Graphs summarize the indexes for (**A**) CL Brener, Berenice, Colombiana and Y strains and (**B**) Y-R^Bz^ parasites. Bars represent the mean + SEM and the values of significance were represented by (*) for *p* < 0.05 and (***) *p* < 0.001, *n* = 9–10 independent experiments.

In order to compare ABCC-like mediated efflux among *T. cruzi* strains, inhibition indexes were calculated for MK-571 inhibitor since it does not inhibit other ABC subfamilies (Figure 7A). The index is calculated as the ratio of the CF MFI in the presence of inhibitor to the one in its absence and indicated the level of ABCC activity. Berenice and CL Brener presented higher index than Y and Colombiana strains and this suggests a greater aptitude for CF efflux in those strains. As a result, ABCC activity was higher in sensitive strains, which indicates an inverse correlation with natural resistance to benznidazole. The index of Y strain was obtained from da Costa et al. [29] and included for the purposes of comparison.

### 2.6. CF Efflux Increases after Acquired Resistance to Benznidazole

Prolonged chemotherapy protocols often lead cells to a drug-adapted phenotype. Although ABCC-like efflux did not relate to natural resistance, exposure to benznidazole would select parasites with a higher capacity for ABCC transport if it were necessary for its survival. The Y strain was selected for prolonged treatment with benznidazole due to its reduced ABCC activity (Figure 7A). Similar to the other strains, efflux of CF on Y-R^Bz^ parasites was inhibited by MK-571 and indomethacin (Figure 6G,H, Supplementary Figure S2G,H). The protocol employed for in vitro selection of resistant parasites led to increases in inhibition index (Figure 7B) in relation to the parental strain, suggesting that ABCC-like activity could participate in the acquired resistance. Remarkably, Y-R^Bz^ was more resistant to the reduction in thiol levels caused by benznidazole and hemin when compared to the Y strain (Figure 2B). These results suggest the influence of thiol biosynthesis pathways on the acquisition of a drug-resistant phenotype.

### 2.7. Resistance to Benznidazole Is Not Directly Dependent on ABCC-Like Efflux

**Figure 8:**
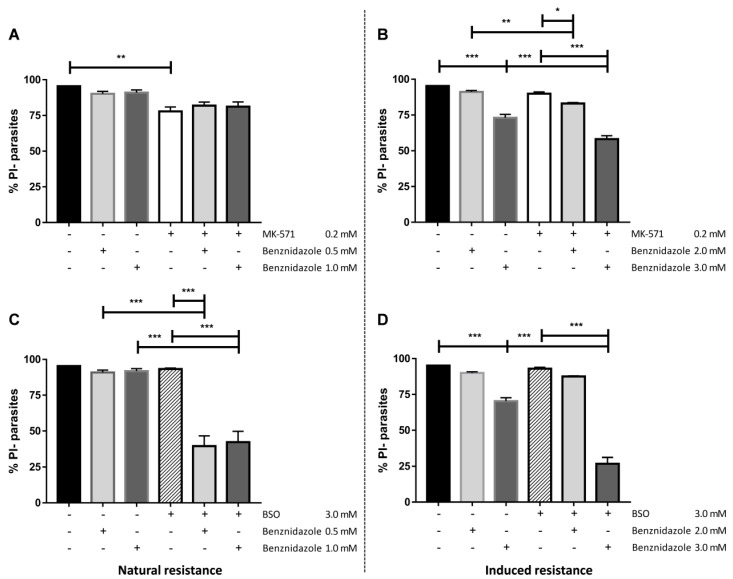
Viability of parasites after benznidazole treatment and thiol depletion. The viability was measured by staining parasites with 1 µg/mL propidium iodide (PI) for 15 min after incubation with benznidazole alone or in combination with 200 µM MK-571 (**A**,**B**) or 3 mM buthionine sulfoximine (BSO) (**C**,**D**) for 24 h. (**A**,**C**) Percentages of PI- parasites (viable cells) from Y strain and (**B**,**D**) Y-R^Bz^ parasites. Bars represent the mean + SEM and the values of significance were represented by (*) for *p* < 0.05, (**) *p* < 0.01, and (***) *p* < 0.001, *n* = 9 (Y) and *n* = 7 (MK-571/Y-R^Bz^) and *n* = 5 (BSO/Y-R^Bz^) independent experiments.

Aiming to assess whether benznidazole could be transported by ABCC, the drug was utilized as a competitor during the CF efflux assay in the Y strain. Our results demonstrate that even in high concentrations, benznidazole was not able to increase the MFI nor the percentages of positive parasites (Supplementary Figure S3). On the other hand, concentrations of 0.5 mM and 1 mM reduced CF MFI values. Next, the effect of inhibition of ABCC activity or thiol depletion on the viability of parasites treated by benznidazole was investigated by vital staining with propidium iodide (PI). The Y-R^Bz^ parasites were less sensitive to ABCC inhibition (MK-571) than the parental strain, with a viability reduction of about 6% in contrast to 18% of the Y strain. The co-treatment with MK-571 and benznidazole did not sensitize the parental Y strain (Figure 8A), but it reduced the viability in Y-R^Bz^ parasites in the highest concentrations of the drug (Figure 8B). Benznidazole and MK-571 had no impact on the percentage of viable Y parasites. However, since roughly 70% of the cells remained viable, these results suggest that *T. cruzi* does not depend exclusively on ABCC-like transport to deal with benznidazole toxicity.

The GSH biosynthesis inhibitor buthionine sulfoximine (BSO) alone produced no effect on the viability of the Y and Y-R^Bz^ parasites (Figure 8C,D). The treatment with benznidazole and BSO promoted a 60% of reduction of the viability on the parental strain, regardless of the concentration of the chemotherapy used. In Y-R^Bz^ parasites, BSO increased benznidazole toxicity in higher concentration of the drug, with a reduction in viability of about 70%. According to results, *T. cruzi* does not depend exclusively on ABCC-like transport to deal with benznidazole toxicity; however, GSH biosynthesis pathway is crucial to neutralize the drug, for which thiol-conjugated intermediates would be extruded by ABCC-like transporters, especially in high concentrations, as part of a complex antioxidative machinery.

## 3. Discussion

Signaling pathways elicited after cellular stress are essential for the survival of *T. cruzi* in diverse microenvironments. They involve the production, transport, and clearance of antioxidants and lipid mediators that often crosstalk with pathways associated to the emergence of chemotherapy resistance. The enzymatic antioxidant machinery in most living cells relies primarily on GSH to reduce and inactivate reactive species. In trypanosomatids, the antioxidant system uses the trypanothione analogue, formed by two GSH molecules linked by one spermidine [32]. Its physicochemical properties and the absence of regeneration enzymes for GSH make *T. cruzi*’s antioxidative route exclusively dependent on di-thiols. This molecule is directly involved in the metabolism of xenobiotics and heavy metals and indirectly in metabolism of peroxides and in regulatory processes [32]. In order to compensate its loss by conjugation or efflux, the trypanothione pool is restored by trypanothione synthase and its oxidized form is regenerated by trypanothione reductase [33]. The transport of GSH can be performed by ABCC proteins, which are involved in cell detoxification and overexpressed in resistant protozoa such as *Leishmania* and *Plasmodium* [25] and in mammals [21]. In this work, we demonstrated that GSH and GSSG modulate the ABCC-like efflux in *T. cruzi* and promote the accumulation of CF. However, it is possible that ABC modulators may not be direct substrates. They are able to bind to the transporter pocket and promote volume and shape changes and stimulate or inhibit their binding to a ligand/substrate, which occurs for verapamil derivatives and certain bioflavonoids [34]. Taking this into consideration, we propose that GSH and GSSG might act as substrates for the ABCC-like activity in *T. cruzi* since we have already demonstrated the direct transport of thiol-conjugated compounds and that the transport of CF can be impaired by thiol depletion and by specific ABCC inhibitors. GSH and GSSG are potentially co-transported with CF since it lacks molecular groups such as maleimide (as in NEM) or chloromethyl (as in CMFDA) that could react with the sulfhydryl radical present in thiols, which would configure a transport in conjugation.

*T. cruzi* does not synthesize heme and must obtain it from external sources [35]. Through the Fenton reaction, heme induces ROS formation and creates a transient oxidative environment that stimulates parasite proliferation [36]. Conversely, metacyclogenesis is favored by a reducing microenvironment provided by antioxidant molecules such as GSH and urate, the latter present in the urine of insect vectors [37]. *T. cruzi* are exposed to a large amount of heme due to the volume of blood ingested by triatomines. In excess, heme is toxic and causes the oxidation of lipids, proteins, and nucleic acids [14]. As a lipophilic anion, it inserts itself into phospholipid membranes, which leads to leakage as a result of changes in permeability and selectivity [38]. Consequently, this selective pressure should have been counteracted by protective adaptations in the parasite. Considering that the Y strain showed ABCC-like activity, we evaluated whether ABCC would participate in protection against heme-induced toxicity. We employed hemin (Fe^+3^ protoporphyrin IX) as a stress-inducing agent, which can be reduced to heme (Fe^+2^ protoporphyrin IX) in reactions involving superoxide, GSH, or ascorbate [39]. Hemin preincubation inhibited CF efflux and this is possibly due to the production of thiol-conjugated intermediates that would be transported by the ABCC-like subfamily members.

ROS are formed as by-products of stress stimuli as well as from normal metabolic processes and affect the metabolism of sphingolipids, such as ceramides [18]. In mammals, they act as coordinators of stress responses, considering that many stress inducers promote accumulation of this sphingolipid, either from sphingomyelin hydrolysis or *de novo* biosynthesis [40]. In *T. cruzi*, the *de novo* pathway is similar to that of mammals up to the formation of ceramides, which are employed for the synthesis of inositolphosphoceramides present in glycoprotein anchors or in free glycosylinositolphospholipids [41]. There are few studies exploring sphingolipids as signaling molecules in protozoa, especially in terms of responses to cellular stress. It is possible that stress induced by cytotoxic agents such as benznidazole or heme promotes the accumulation of ceramides in the parasite in a similar method to mammalian cells. Early accumulation could occur via the remodeling of inositolphosphoceramides, which is possibly induced by GSH depletion [42]. This hypothesis can be supported by other protozoan parasites such as *Plasmodium falciparum*, in which stress induced by the chemotherapeutic agents artemisinin and mefloquine induced ceramide accumulation in a GSH-dependent manner [43]. Another alternative route would be derived from *de novo* biosynthesis, since tamoxifen, an inhibitor of ceramide glycosylation and hydrolysis in humans [44], inhibited inositolphosphoceramide synthase in *Leishmania amazonensis* [45]. Tamoxifen is an oral drug used in the breast cancer treatment and interferes with several cell pathways [46], including sphingolipid metabolism. It has been efficient against species of *Leishmania* both in vivo and in vitro [47].

ABC proteins transport lipids, including phospholipids and sphingolipids [48]. ABCC members were directly implicated in the transport of sphingosine-1-phosphate [22], glucosylceramide, and sphingomyelin in mammalian cell lines [23] in a GSH-dependent manner. Assuming that ceramides could be induced in response to stress in *T. cruzi*, an ABCC-like transporter could regulate the content of these sphingolipids. Preincubation with sphingosine, employed to stimulate de novo biosynthesis of endogenous ceramides, promoted CF accumulation in Y strain. In order to investigate direct transport, we used a fluorescent ceramide analogue as substrate for the efflux assay. Furthermore, we observed that the transport of synthetic short-chain ceramides was inhibited by MK-571 as well as by the depletion of ATP and of free thiols; this demonstrates, for the first time, that ceramides are direct substrates of ABCC-like transport in *T. cruzi*.

Bearing in mind that ABCC transporters have evolved as adaptive advantages for dealing with cytotoxic intermediates of diverse cell processes, we evaluated whether the ABCC activity could relate to cellular protection in naturally or in benznidazole-induced resistant strains. All strains exhibited ABCC-like activity; however, the naturally sensitive ones showed higher inhibition indexes which indicates higher activity. For that reason, it does not seem that ABCC efflux would be associated to natural resistance. Resistance to benznidazole in *T. cruzi* can result from different mechanisms that mainly involve (i) the regulation of the activation pathways of the prodrug, (ii) the defense pathways against free radicals, and (iii) the increase in the efflux of the drug. Therefore, other intrinsic factors could explain the Y and Colombiana resistant phenotype, such as the overexpression of enzymes of the trypanothione/GSH biosynthesis pathway and mutations in nitroreductases genes, which participates in benznidazole metabolism [49,50].

The acquired resistance was achieved in the Y strain by prolonged exposure to benznidazole and it showed the lowest ABCC-like activity. Y-R^Bz^ parasites showed higher IC_50_ for the drug and an increase in ABCC efflux when compared to the parental strain. ABCB1-like activity was analyzed by efflux of fluorescent substrate rhodamine 123 (Rho 123) in the presence of known pharmacological inhibitors: cyclosporine A and verapamil. As noted for strain Y [29], ABCB1-like activity does not influence natural or acquired resistance, since no strain presented ABCB1-like efflux (Supplementary Figure S4) which suggests the absence of a functional transporter. ABCC-like proteins did not transport benznidazole directly because the drug was not able to increase the CF accumulation even in high concentrations. Despite that, Y-R^Bz^ parasites showed resistance to the reduction of thiol levels either by drug or hemin administration, which indicates an adaptation of the GSH biosynthesis pathway for the acquisition of the resistant phenotype. The effect of ABCC inhibition and GSH depletion showed interesting effects for benznidazole toxicity. The ABCC inhibition (MK-571) did not affect the viability on the Y strain treated with subtoxic concentrations of benznidazole. Nevertheless, MK-571 and benznidazole presented additive effects in reducing the population of viable Y-R^Bz^ parasites. The inhibition of GSH biosynthesis (BSO) had a relevant impact for the response to drug-induced stress in the parental strain, with significant reduction of viability in co-treatment. Y-R^Bz^ parasites tolerated the stress of GSH depletion and of benznidazole treatment to a certain extent; however, the co-treatment in the higher concentration of benznidazole reduced the viability to levels comparable to the Y strain, effectively reversing the resistant phenotype. We suggest that ABCC-like transport can perform the efflux of benznidazole metabolites conjugated to GSH, thus explaining its increase in acquired resistance. One possible candidate for this would be glyoxal, which is a cytotoxic metabolite generated after benznidazole reduction by a type I nitro-reductase in *T. cruzi* that interacts with reduced thiols [10]. It appears that *T. cruzi* mobilizes redundant mechanisms to deal with the intermediates from benznidazole metabolization, since inhibition of ABCC-like activity had little effect on drug toxicity. In high concentrations of benznidazole, the ABCC transport seems to be more important in reducing toxicity for Y-R^Bz^ parasites and this is likely due to the exhaustion of the other routes of the antioxidative machinery.

Faundez et al. demonstrated that thiol biosynthesis was important for resistance to chemotherapy once treatment with BSO had increased the toxicity of benznidazole and nifurtimox, which is another chemotherapeutic possibility for Chagas disease [51,52]. Studies have shown that antioxidant enzymes such as tryparedoxin peroxidase [53] and iron-superoxide dismutase-A [54] participate in the protection to reactive species produced from benznidazole metabolism. In contrast, participation of transporters involved in the response to xenobiotics had been minimally explored. ABC transporters could work in tandem on a resistant phenotype, taking into consideration that *T. cruzi* presents 27 ABC genes and four belonging to that of the ABCC subfamily. The ABCG1 gene was found to be overexpressed in strains naturally sensitive to benznidazole in *T. cruzi* [55,56], but its functionality has not been studied so far. Identifying the specific transporter responsible for the thiol efflux would greatly contribute to the understanding of the acquisition of resistance and ultimately further the efforts of drug development towards Chagas disease. Since we are aware of this, focus should be concentrated on producing knockout clones for the ABC subfamilies, which in association with efflux assays in presence of pharmacologic modulators, will allow us to achieve these objectives.

In conclusion, benznidazole and hemin are able to produce thiol-conjugated intermediates and to activate the synthesis and remodeling of ceramides, which act as mediators of cellular stress pathways. Our results bring to light the processes mediating *T. cruzi* adaptation to natural or xenobiotic stresses, in which efflux transporters such as ABC proteins and markedly the ABCC subfamily perform the role they accomplish best: reducing cytotoxicity by efflux of xenobiotics or metabolites and managing levels of mediators of cell death in coordination with the GSH pathway.

## 4. Materials and Methods

### 4.1. Cultures of Trypanosoma Cruzi Strains

CL Brener, Berenice, and Colombiana strains of *T. cruzi* were donated by Dr. Policarpo A. Sales Junior of the Rene Rachou Research Center of the Fundação Oswaldo Cruz (FIOCRUZ) from Minas Gerais, Brazil. The Y strain was kindly donated by Professor Celio Freire-de-Lima from the Institute of Biophysics Carlos Chagas Filho (IBCCF) of the Universidade Federal do Rio de Janeiro (UFRJ), Rio de Janeiro, Brazil.

Epimastigote forms were cultivated at 27 °C in Brain and Heart Infusion medium (BHI, BD Biosciences, São Paulo, SP, Brazil) supplemented with 10% fetal bovine serum (FBS, Life Technologies of Brazil, São Paulo, SP, Brazil), 20 μg/mL folic acid (Sigma-Aldrich, São Paulo, SP, Brazil), 12.5 μg/mL hemin (Sigma-Aldrich), and 50 μg/mL gentamicin (Sigma-Aldrich). For subcultures, epimastigote forms were collected weekly and 10^6^ parasites/mL were suspended in complete BHI medium. All centrifugations were performed at 1000× *g* for 10 min at room temperature.

### 4.2. MTT Reduction Assay

The tetrazolium salt MTT (3-(4,5-dimethylthiazol-2-yl)-2,5-diphenyltetrazolium bromide) was used to assess mitochondrial reducing activity. During respiration, cells convert the water-soluble MTT to the insoluble purple product formazan, which is solubilized in DMSO and its concentration determined by optical density. Briefly, the amount of 10^6^ epimastigotes/mL was distributed on 96-well culture plates in complete BHI medium and benznidazole was added to final concentrations from 0.001 mM to 1 mM. After 48 h, plates were centrifuged and the supernatants discarded, followed by the addition of PBS supplemented with 2 g/L glucose and 10% FBS. Plates were incubated for 4 h at 27 °C in the dark after adding a solution 2.5 mg/mL MTT and 0.22 mg/mL phenazine methosulfate (both from Sigma-Aldrich). Next, plates were centrifuged and the supernatants discarded. Formazan crystals were dissolved in DMSO (Sigma-Aldrich) and plates were shaken for 20 min at room temperature protected from light. Absorbance reading was performed at 570 nm on a Beckman Coulter AD340 spectrophotometer (Beckman Coulter, Brea, CA, USA). Experiments were performed in triplicate and IC_50_ of mitochondrial reducing activity after exposure to benznidazole was determined by logarithmic regression of the normalized percentage curve using the GraphPad Prism software (version 7.0, GraphPad, San Diego, CA, USA). Benznidazole was provided by Dr. Nubia Boechat Andrade of Institute of Technology in Pharmaceuticals of the Oswaldo Cruz Foundation from Rio de Janeiro, Brazil.

### 4.3. In Vitro Induction of Resistance to Benznidazole

The amount of 10^6^ epimastigotes/mL of the Y strain was maintained in complete BHI medium with the addition of 30 μM benznidazole (IC_50_ obtained after MTT assays) at 27 °C. After 2 days, the parasites were centrifuged, supernatants discarded, and complete BHI medium added to allow replication of the surviving parasites for the next 5 days. Parasites were submitted to the same procedure up to 8 weeks. Then, the concentration of benznidazole was increased in 10 µM per week up to a final concentration of 120 µM. Resistance to benznidazole was then evaluated by the MTT reduction assay as described before. Thereafter, the benznidazole-adapted Y strain was named Y-R^Bz^. For subcultures, the amount of 10^6^ epimastigotes/mL was suspended in complete BHI medium containing 120 µM benznidazole for 7 days.

### 4.4. ATP Depletion

The irreversible inhibition of glyceraldehyde-3-phosphate-dehydrogenase by alkylating agents as IAA reduces glycolysis and, consequently, ATP levels [57]. For this, the amount of 10^7^ epimastigotes/mL was incubated in absence or presence of 2 mM IAA (Sigma-Aldrich) in PBS for 1 h at 27 °C [58]. Next, parasites were centrifuged, supernatants discarded, and parasites were suspended in PBS for the ABC-mediated efflux assay. In this case, PBS was employed instead of RPMI during the efflux assay due to absence of glucose.

### 4.5. ABC-Mediated Efflux Assay

The efflux assay was divided into 30 min steps: accumulation and efflux of substrate [59]. For the ABCC-mediated efflux assay, the CFDA dye (Life Technologies of Brazil) was employed. In the cytosol, CFDA is hydrolyzed and is what originates the fluorescent substrate CF, which is transported to extracellular medium by ABCC subfamily members. Briefly, the amount of 10^7^ epimastigotes/mL was incubated with 50 μM CFDA diluted in RPMI medium (Sigma-Aldrich) in the absence or presence of 600 µM indomethacin or 200 µM MK-571 as ABCC inhibitors (both from Sigma-Aldrich). After the accumulation step, parasites were centrifuged and then suspended in RPMI medium in the absence or presence of the inhibitors. Afterwards, parasites were centrifuged, supernatants discarded, and parasites suspended in PBS supplemented with 5% FBS and kept on ice for immediate acquisition by flow cytometry.

Alternatively, 5 mM GSH, 5 mM or GSSG (all from Sigma-Aldrich), or 0.5, 1.0, 2.0 or 3.0 mM benznidazole were employed as competitive inhibitors in the same manner. Otherwise, parasites were preincubated with 200 µM hemin or 60 µM sphingosine (all from Sigma-Aldrich) for 1 h and removed before the assay.

For ceramide efflux, 10 µM C6-NBD-cer (Avanti Polar Lipids, Alabaster, AL, USA) was employed as fluorescent substrate in the absence or presence of 200 μM MK-571 in RPMI.

Similarly, the naturally fluorescent substrate Rho 123 was employed to analyze ABCB1-mediated efflux assay. Therefore, epimastigote forms were incubated with 100 nM Rho 123 (Sigma-Adrich) diluted in RPMI medium in absence or presence of 50 μM Cyclosporin A and 10 μM Verapamil (Sigma-Aldrich) as ABCB1 inhibitors in the accumulation and efflux steps. CsA was kindly donated by Dr. Marcia Capella from the IBCCF and UFRJ, Rio de Janeiro, Brazil. The efflux assays were performed at 27 °C. The transport inhibition index was calculated as the ratio of the MFI in the presence of inhibitor to the one in the absence, suggestive of level of ABC activity. As negative control, parasites were not exposed to dyes and the efflux assays in the epimastigote forms were performed at 27 °C. Multidrug resistance cells Lucena-1 or FEPS were employed as positive control due to the overexpression of ABCB1 and ABCC1, respectively. Dr. Vivian Rumjanek from the Institute of Medical Biochemistry Leopoldo de Meis, UFRJ, Rio de Janeiro, Brazil gently offered these cells.

### 4.6. Determination of Intracellular Thiols

The amount of 10^7^ epimastigotes/mL was incubated in the absence (CTL) or presence of 0.5 or 1.0 mM benznidazole or 0.2 mM hemin in PBS supplemented with 2 g/L glucose for 3 h at 27 °C. Afterwards, parasites were centrifuged, supernatants discarded and parasites incubated in PBS containing 1.5 µM CMFDA at 27 °C for 15 min prior to acquisition by flow cytometry [58]. The assay was performed in the absence of glucose and cells were maintained on ice until acquisition in the flow cytometer to minimize interference of ABCC-like efflux. CMFDA is an acetoxymethyl ester derivative and is able to cross the plasma membrane efficiently. In the cytosol, it reacts with exposed sulfhydryl radicals, forming TMF. As the negative control, parasites were not exposed to the dye. As positive control of thiol depletion, parasites were incubated in PBS containing 0.1 mM NEM, an alkylating agent, for 1 h and removed before addition of CMFDA.

### 4.7. Assessment of Cellular Viability

The staining of nonviable parasites was performed by the DNA intercalation dye PI, which is readily excluded by live cells with intact membranes. The amount of 10^7^ epimastigotes/mL was incubated at 27 °C for 24 h with concentrations ranging from 1.0 to 3.0 mM benznidazole diluted in complete BHI medium in the absence or presence of 200 µM MK-571 or 3 mM BSO (buthionine sulfoximine), which is an irreversible inhibitor of GSH biosynthesis. Parasites were then centrifuged, supernatants discarded, and parasites suspended in 1 µg/mL PI diluted in PBS and incubated for 15 min prior to acquisition by flow cytometry. As the positive control of cell death, cells were incubated with distilled water for 30 min before addition of dye. As autofluorescence control, parasites were not exposed to the dye.

### 4.8. Flow Cytometry Analyses

The CF, TMF, and C6-NBD-cer fluorescence intensities were acquired on the FL1-H channel (530/30 bandpass filter), while PI fluorescence were acquired on the FL3-H channel (670LP filter) of a FACSCalibur (BD Biosciences, San Jose, CA, USA). Post-analysis was performed in the software Summit (version 4.3, Dako Colorado, Fort Collins, CO, USA) on at least 10,000 viable cells that were gated in accordance with forward (FSC) and side scatter (SSC) parameters representative of cell size and granularity. Median fluorescence intensities (MFI) data and percentages of parasites were acquired from histograms for each dye. A negative/low fluorescence gate was designed containing 95% of control parasites from the histogram origin while a high fluorescence gate contained the remaining.

### 4.9. Statistical Analysis

Statistical analyses were performed using the software GraphPad Prism. For two nonpaired comparisons, the t-student or Mann-Whitney tests were respectively employed for parametric and nonparametric data. For more than two comparisons, one-way ANOVA or Kruskal–Wallis tests were respectively employed for parametric and nonparametric data. For paired nonparametric data, the Wilcoxon or Friedman tests were performed for two or more than two comparisons. The Tukey’s, Sidak’s, Dunn’s, or Dunnet’s post-tests were used according to the compared columns. Significance values were represented by (*) for *p* < 0.05, (**) for *p* < 0.01, and (***) for *p* < 0.001.

## Data Availability

All the data are contained within the article and Supplementary Materials.

## Supplementary Materials

The following are available online, Figure S1: IC_50_ for benznidazole in *T. cruzi* strains and Y-R^Bz^ parasites; Figure S2: CF accumulation in the presence of the inhibitor indomethacin in *T. cruzi* sensitive and resistant to benznidazole; Figure S3: CF accumulation in the presence of benznidazole in *T. cruzi*; Figure S4: Rho 123 accumulation the presence of ABCB1 inhibitors in *T. cruzi* sensitive or resistant to benznidazole.

## Sample Availability

Samples of the compounds are available commercially.

## Abbreviations

Δ	inhibition index of transport
ABC	ATP-binding cassette
ABCB1	subfamily B, member 1 (P-glycoprotein)
ABCC	subfamily C (MRP)
ABCG1	subfamily G, member 1
BSO	buthionine sulfoximine
Bz	benznidazole
C6-NBD-ceramide	*N*-[(*E*,2*S*,3*R*)-1,3-dihydroxyoctadec-4-en-2-yl]-6-[(4-nitro-2,1,3-benzoxadiazol-7-yl)amino]hexanamide
CF	carboxyfluorescein
CFDA	5(6)-carboxyfluorescein diacetate
CMFDA	5-chloromethylfluorescein diacetate
GSH	reduced glutathione
GSSG	glutathione disulfide form (oxidized glutathione)
IAA	Iodoacetic acid
MK-571	(*E*)-3-[[[3-[2-(7-chloro-2-quinolinyl)ethenyl]phenyl][[3-(dimethylamino)-3-oxopropyl]thio]methyl]thio]-propanoic acid
MFI	median fluorescence intensity
MTT	3-(4,5-dimethylthiazol-2-yl)-2,5-diphenyltetrazolium bromide
NEM	*N*-ethylmaleimide
PI	Propidium iodide
Rho 123	rhodamine 123
ROS	reactive oxygen species
TMF	thiol-conjugated methylfluorescein
