# Supplementary material for: Intrinsic and Chemotherapeutic Stressors Modulate ABCC-Like Transport in Trypanosoma cruzi
Source: Molecules. 2021 Jun 9;26(12):3510. doi: 10.3390/molecules26123510 (PMC8227891; doi:10.3390/molecules26123510)
Supplement: Supplementary file 1 [file molecules-26-03510-s001.zip › molecules-1237611-supplementary.pdf]

Supplementary Materials

Intrinsic and Chemotherapeutic Stressors Modulate ABCC-Like Transport in *Trypanosoma cruzi*

Kelli Monteiro da Costa [1,*], Eduardo J. Salustiano [1], Raphael do Carmo Valente [2], Leonardo Freire-de-Lima [1], Lucia Mendonça-Previato [1,*] and José Osvaldo Previato [1]

1 Laboratory of Glycobiology, Carlos Chagas Filho Institute of Biophysics, Federal University of Rio de Janeiro (UFRJ), Rio de Janeiro 21941-902, RJ, Brazil; salustiano@bioqmed.ufrj.br (E.J.S.); leolima@biof.ufrj.br (L.F.L.); previato@biof.ufrj.br (J.O.P.)

2 Nucleus of Multidisciplinary Research in Biology (Numpex-Bio), UFRJ Duque de Caxias Campus—Professor Geraldo Cidade, Federal University of Rio de Janeiro (UFRJ), Duque de Caxias 25250-470, RJ, Brazil; raphael.valente@xerem.ufrj.br

* Correspondence: kellimc85@biof.ufrj.br (K.M.C.); luciamp@biof.ufrj.br (L.M.P.)

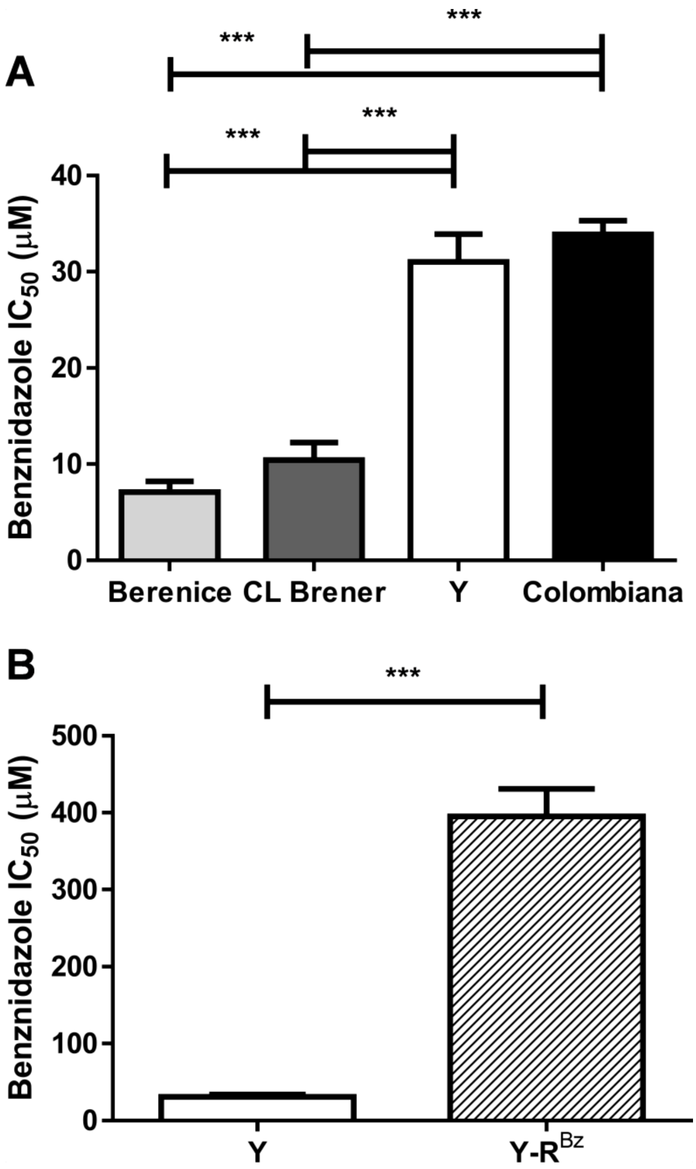

Figure S1. IC_{50} for benznidazole in *T. cruzi* strains and Y-R^{Bz} parasites. In MTT assay, the half-maximum inhibitory concentration (IC_{50}) of mitochondrial reducing activity was determined from logarithmic regression as described in Material and Methods in the CL Brener, Berenice, Colombiana and Y strains and Y-R^{Bz} parasites. Bars represent the mean + SEM. Significance values were represented by (***) for $p < 0.001$, $n = 4$ (Colombiana) and $n = 6$ (others) independent experiments.

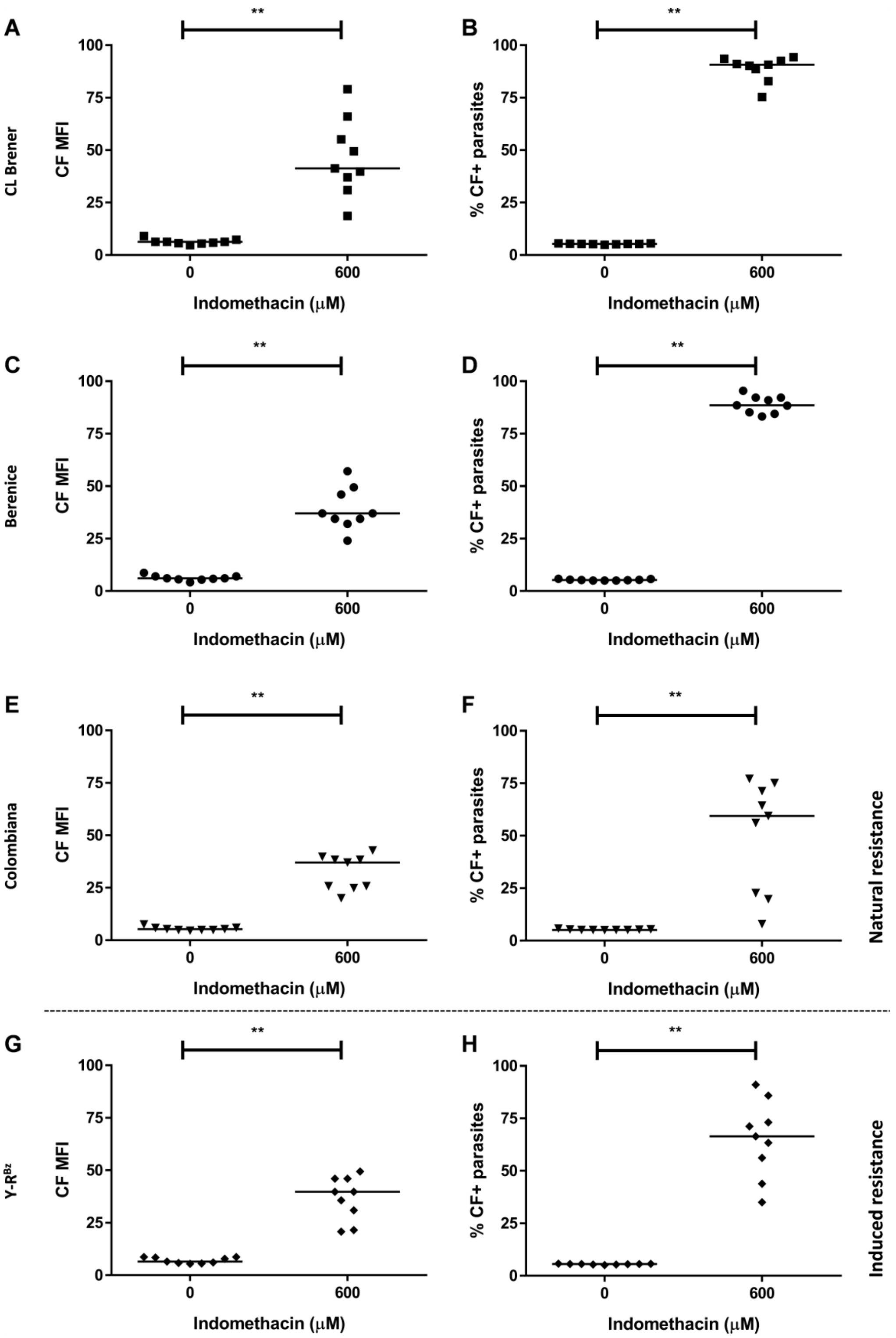

Figure S2. CF accumulation in the presence of the inhibitor indomethacin in *T. cruzi* sensitive and resistant to benznidazole. ABCC-like activity was evaluated by the CF efflux assay in the presence of 600 μM indomethacin. Graphs exhibit CF MFI (left panel) and percentage of CF+ parasites (right panel) from (**A** and **B**) CL Brener, (**C** and **D**) Berenice, (**E** and **F**) Colombiana strains and (**G** and **H**) Y-R^{Bz} parasites. Lines represent the median and values of significance were represented by (**) for $p < 0.01$ from $n = 9$ independent experiments.

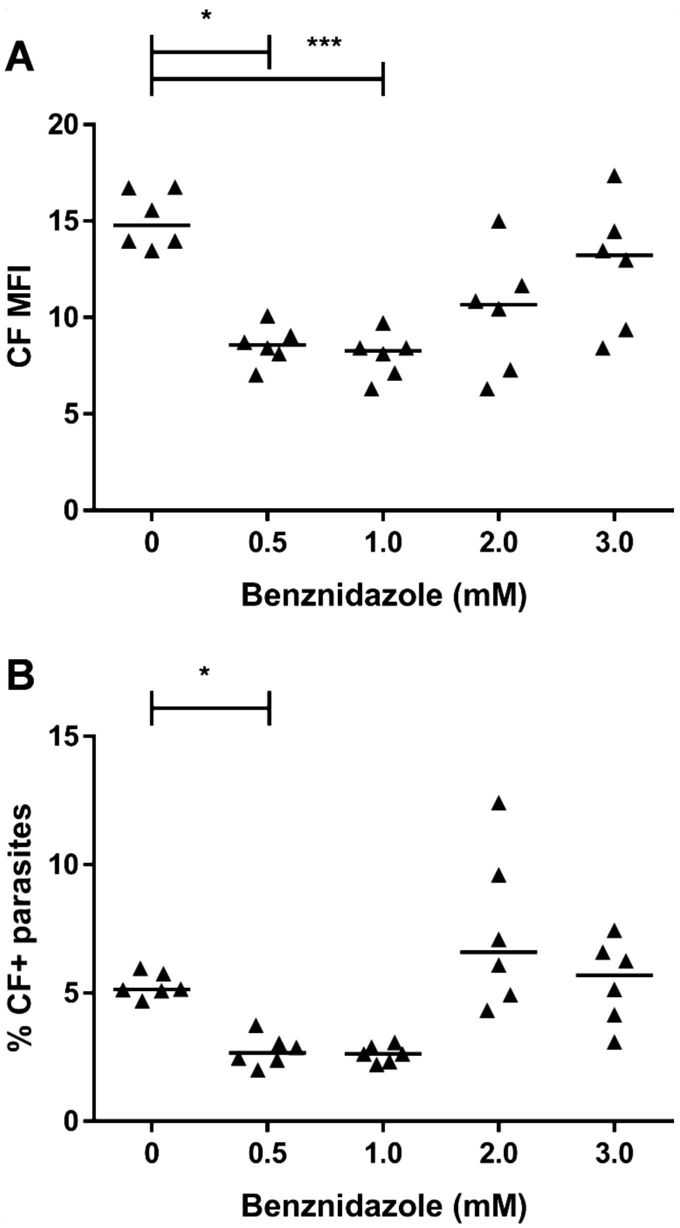

Figure S3. CF accumulation in the presence of benznidazole in *T. cruzi*. ABCC-like activity was evaluated by the CF efflux assay in the presence of benznidazole. Graphs represent (**A**) the CF MFI and (**B**) percentage of CF+ parasites in the Y strain. Lines represent the median and values of significance were represented by (*) for $p < 0.05$ and (***) $p < 0.001$, $n = 6$ independent experiments.

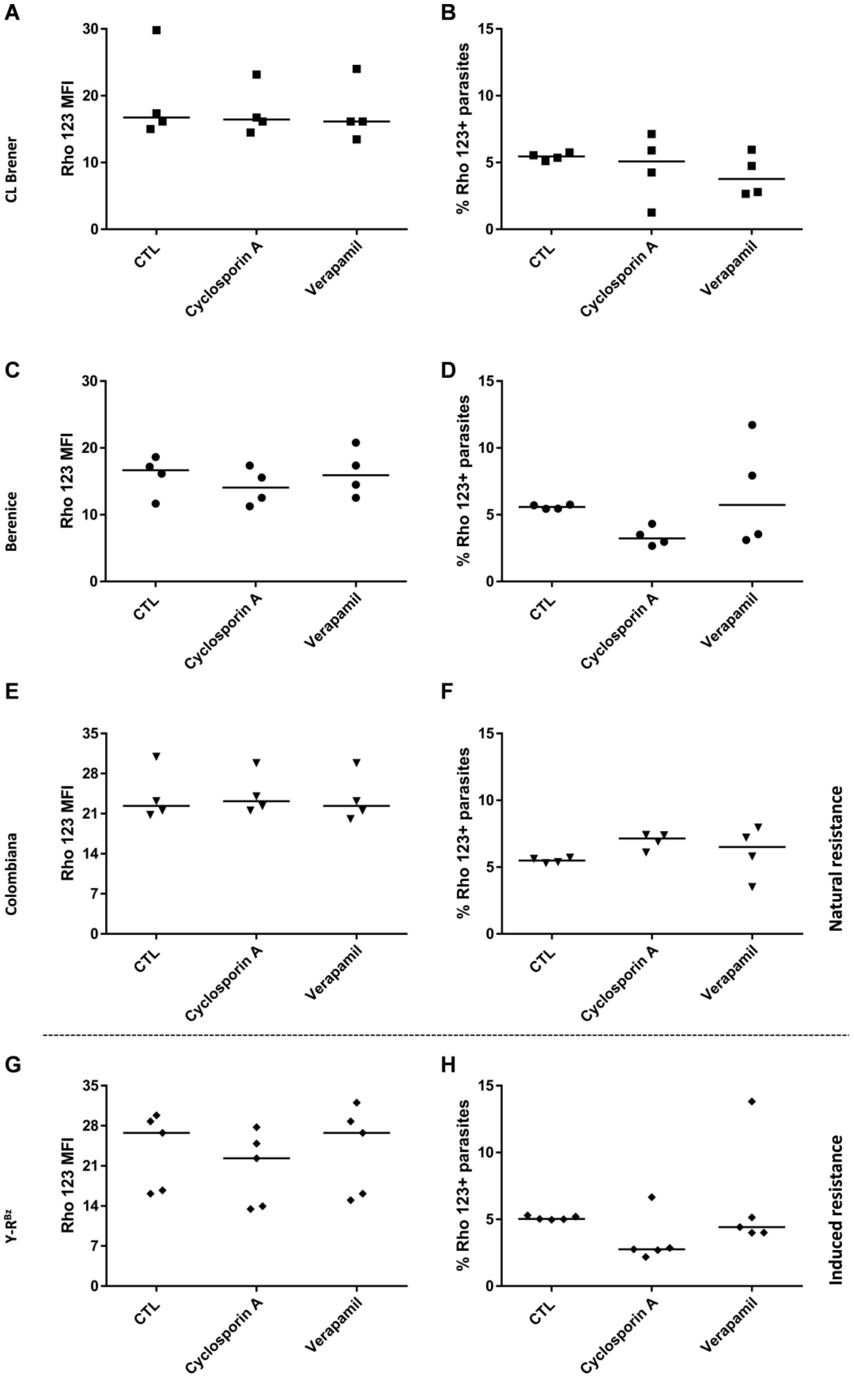

Figure S4. Rho 123 accumulation the presence of ABCB1 inhibitors in *T. cruzi* sensitive or resistant to benznidazole. ABCB1-like activity was evaluated by rhodamine 123 (Rho 123) efflux assay in the presence or absence of 50 µM cyclosporin A or 10 µM verapamil. Graphs exhibit Rho 123 median fluorescence intensity (MFI) (left panel) and percentage of CF+ parasites (right panel) for epimastigote forms from (**A** and **B**) CL Brener, (**C** and **D**) Berenice and (**E** and **F**) Colombiana strains and (**G** and **H**) Y-R^{Bz} parasites. Lines represent the median and values of significance were represented by (*) for $p < 0.05$ and (**) $p < 0.01$, $n = 4$ (strains) and $n = 5$ (Y-R^{Bz}) independent experiments.
